# Smart Cells Against Cancer: Advances in Cell-Based Drug Delivery and Diagnostics

**DOI:** 10.3390/pharmaceutics18010028

**Published:** 2025-12-25

**Authors:** Lisa Gherardini, Giovanni Inzalaco, Sara Gargiulo, Lorenzo Franci, Monia Taranta

**Affiliations:** 1 Istituto di Fisiologia Clinica (IFC), Consiglio Nazionale delle Ricerche (CNR), 53100 Siena, Italy; sara.gargiulo@cnr.it (S.G.); lorenzofranci@cnr.it (L.F.); 2 Istituto degli Endotipi in Oncologia, Metabolismo ed Immunologia (IEOMI), Consiglio Nazionale delle Ricerche (CNR), 80131 Napoli, Italy; giovanniinzalaco@cnr.it

**Keywords:** drug delivery, cellular carrier, cancer, glioblastoma

## Abstract

Cell-based drug delivery has emerged as a powerful strategy to improve therapeutic targeting while reducing systemic toxicity. This approach is particularly valuable for anticancer agents, which are often limited by severe side effects arising from off-target activity and non-specific distribution. By using cells as carriers, drugs can evade immune clearance, achieve prolonged circulation, and improve pharmacokinetic profiles, ultimately enhancing therapeutic efficacy. This review surveys the current landscape of cell-mediated drug delivery in oncology, emphasizing both fundamental principles and practical applications. We discuss the design and preparation of cellular carriers, examine the unique characteristics of commonly used cell types, and highlight recent technological innovations that are expanding their theranostic potential, focusing on strategies for delivery to challenging anatomical sites, with a dedicated focus on the brain. By consolidating recent advances and insights, this review aims to provide a comprehensive perspective on the promise and future directions of cell-based drug delivery for cancer therapy.

## 1. Introduction

Cancer remains one of the most critical health challenges on a global scale, with 20 million new cases reported in 2022 and nearly 35 million projected by 2050, according to the International Agency for Research on Cancer [1]. Despite advances in therapy, cancer is still a leading cause of mortality, emphasizing the urgent need for more effective treatments. While systemic therapies can be potentially effective against cancer, their clinical utility is often hindered by low bioavailability and poor specificity, often requiring high doses that cause severe off-target toxicity [2]. In this context, the development of targeted treatment strategies is a critical priority.

Drug-delivery systems (DDS) capable of directing therapeutic agents to the disease site while minimizing off-target release offer a promising solution. Such systems can improve the efficacy of both conventional and novel drugs and enhance safety and tolerability by enabling lower or controlled dosing, reducing systemic exposure and side effects, and ultimately improving treatment outcomes [3,4]. This is particularly relevant given the high cost, long timelines, and low success rates of new drug development, with only 10–20% of clinical candidates achieving approval [5].

An ideal DDS should be biocompatible, protect drugs from rapid clearance by the mononuclear phagocytic system (MPS), and enable precise targeting or sustained release [6]. In this review, we focus on cell-based DDS, as they possess the necessary qualities to meet all the listed criteria, offering relevant advantages over other delivery systems. Cells are inherently biocompatible and biodegradable, and the use of autologous or blood-type-matched material minimizes immune responses [7]. Surface molecules such as CD47, known as the “don’t eat me” signal, reduce rapid clearance by macrophages, thereby prolonging drug half-life and enhancing therapeutic efficacy. Moreover, cells offer high cargo capacity, enabling the transport of substantial amounts of therapeutic agents, which can be either encapsulated intracellularly or loaded onto the cell membrane. Due to the versatility of these carriers in transporting different types of drugs, they can be used to address a wide range of pathologies. This review concentrates on the application of cell-based DDS in cancer therapy. We begin by outlining the fundamental principles for designing cellular carriers, covering targeting strategies, drug loading approaches, and release mechanisms for the most utilized cell types, emphasizing their roles in tumor treatment. While our primary attention is turned on whole-cell systems and their distinctive advantages, we also briefly mention some cell-derived structures, such as membranes and isolated vesicles, which have attracted widespread interest as versatile, bioinspired platforms for drug delivery. To provide a solid foundation, we draw on key contributions from both classic and contemporary literature that established the basis for the characterization and use of these DDS. Building on this groundwork, we then turn to a particularly challenging malignancy, glioblastoma, for which we highlight concrete advances in the preclinical research field and in the clinical trials from the past three years and discuss the current opportunities and remaining hurdles in applying cellular carriers against this aggressive tumor.

## 2. Cell-Based Drug Delivery Systems

### 2.1. Targeting Strategies

#### 2.1.1. Passive Targeting

**Figure 1:**
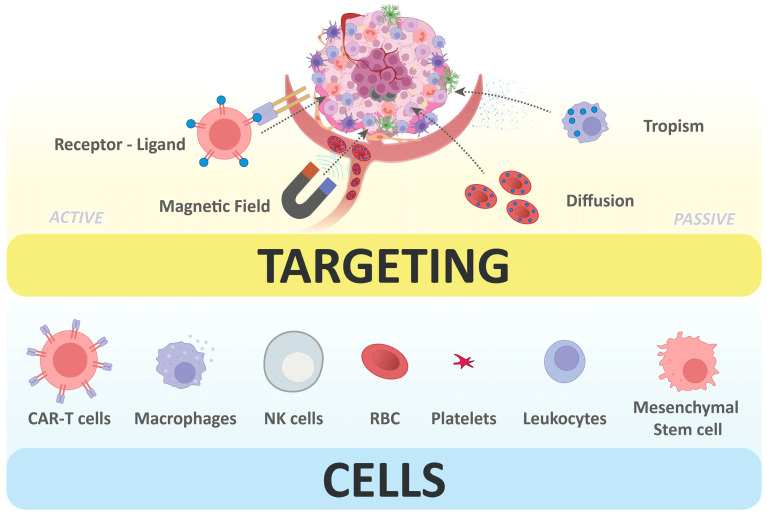
Cell-based drug delivery systems and targeting. Overview of cell types employed as carriers for cell-based drug delivery systems (DDSs), including mesenchymal stem cells, immune cells (leukocytes, lymphocytes, and neutrophils), chimeric antigen receptor T (CAR-T) cells, natural killer (NK) cells, and macrophages. Red blood cells and platelets are also utilized as systemic carriers. Passive targeting exploits the natural circulatory behavior or intrinsic tissue tropism of carrier cells, whereas active targeting is achieved via receptor–ligand interactions or by external magnetic guidance of iron nanoparticle-loaded cells.

Strategies to deliver therapeutics to tumor sites can rely on either passive or active targeting (Figure 1). In cell-based DDS, passive targeting exploits the cells’ homing ability, which refers to their intrinsic tendency to accumulate in certain organs or tumors because of a specific tropism [8,9]. The tumor microenvironment is typically characterized by hypoxia, acidosis, and elevated levels of pro-inflammatory cytokines and pro-angiogenic factors. These act as chemoattractants, driving the recruitment and migration of multiple cell types, particularly, but not exclusively, immune cells. Macrophages, monocytes, neutrophils, T cells, natural killer cells, and mesenchymal stem cells display homing capabilities, either toward metastatic niches or directly to primary tumor sites [10,11]. Numerous studies have demonstrated that the natural tumor-tropism of some cells can be harnessed to accumulate anticancer therapeutics at the disease site [12,13,14,15].

Interestingly, cancer cells themselves also possess homing ability. Circulating metastatic cells have been observed to return not only to secondary sites but also home of the primary tumor [16]. This phenomenon has inspired strategies employing tumor cells as carriers to deliver therapeutics to both metastatic and primary lesions [17].

Among all cell types, red blood cells (RBCs or erythrocytes) are the most extensively studied for DDS applications. The bloodstream is their natural environment, and under normal, non-pathological conditions, RBCs typically do not extravasate or exhibit a particular affinity for specific organs. An exception is the MPS, which includes specialized compartments primarily present in the spleen and liver that allow the passage of RBCs and other particles for the clearance of foreign substances as well as senescent or damaged cells. This makes these organs potential targets for passive RBC-mediated drug delivery [7].

#### 2.1.2. Active Targeting

In some cases, active targeting is preferred because it can substantially increase drug delivery to the intended site and further reduce toxicity associated with the limited selectivity of passive targeting. This strategy aims to enhance carrier affinity for target cells, promoting selective payload accumulation at disease sites while minimizing off-target interactions.

A common approach involves modifying carrier membranes with ligands that recognize cell surface markers ideally unique to or overexpressed by tumor cells, the surrounding tissue, or the tumor microenvironment. Examples include folate receptors [18], epidermal growth factor and vascular endothelial growth factor receptors [19], integrins [20], and others [21]. Recent advancements in biomaterials and nanotechnology have expanded methods for conjugating ligands onto biological membranes, making active targeting increasingly feasible. Furthermore, the development of click chemistry has provided a variety of bio-orthogonal reactions that can be exploited for this purpose, providing enhanced specificity and biocompatibility [22]. In particular, copper-free bio-orthogonal reactions, pioneered by Bertozzi and colleagues [23], are well-suited to biological systems due to their efficiency under physiological conditions, high specificity, and low cytotoxicity [24]. Typically, these reactions proceed in two steps. First, metabolic engineering introduces bio-orthogonal groups, such as azides or N-methacryloyl mannosamine, into cell membrane components. Second, complementary groups on nanoparticles, biomolecules, or small-molecule drugs selectively couple with these labels, enabling precise and biocompatible surface modification [25]. Table 1 provides some examples of surface modifications used for active tumor targeting by cell-based DDS.

These approaches hold considerable promise and warrant further investigation. Nonetheless, careful evaluation is necessary to ensure their efficacy and safety, as chemical modifications involving membrane proteins may impair their function, and the insertion of lipid chains into membranes could alter physiological properties, potentially compromising immune evasion and accelerating clearance.

Beyond ligand-based strategies, other approaches, such as loading carriers with magnetic nanostructures, especially superparamagnetic iron oxide NPs (SPIONs), have also been explored. Under an external magnetic field, these carriers exhibit enhanced localization and retention at target sites while avoiding extensive chemical modification of the cell surface [12,26,27,28].

Overall, active targeting represents a powerful and promising strategy. The delivery systems must first reach and access the target site of interest to have the chance of establishing a specific interaction. Thus, overcoming biological barriers and ensuring sufficient passive delivery remain essential, even when employing active targeting.

**Table 1:** Examples of cell-based DDS surface modifications for active targeting of tumors.

Type	Ligand on DDS Surface	Linking Strategy	Experimental Model	Refs.
RBCs	Folate	DSPE-PEG-folate	4T-1 cells; in vivo breast model	[29]
	Nucleolin-binding aptamer	Lipid-PEG-aptamer	KB cells	[30]
	RGD peptide	DSPE-PEG-streptavidin-biotin-PEG-RGD	In vivo glioma model	[20]
	Hyaluronidase enzyme	NHS-PEG-rHuPH20	PC3 cells	[31]
	Anti-PECAM and anti-ICAM antibodies	Dual-targeted liposomes	Mouse lungs; ex vivo human lungs	[9]
	Anti-EpCam antibody	DSPE-PEG-biotin-avidin-biotinylated Ab	4T1 cells	[32]
	Mannose	DSPE-PEG-mannose	DC2.4 cells; in vivo melanoma model	[33]
	DWSW and NGR	DSPE-PEG-DWSW DSPE-PEG-NGR	bEnd.3, HUVEC, C6 cells; in vivo glioma model	[34]
NK cells	CD22 ligand	Sialic acid biosynthetic pathway	Raji cells; patient-derived lymphoma cells; in vivo lymphoma model	[35]
RAW264.7	PTK7-binding aptamers	ManM/SH-	CCRF-CEM cells	[36]
MSC exosomes	Sgc8 aptamer	Sgc8-COOH/NH2-	B16F0 cells; in vivo melanoma model	[37]
MC membranes	RGD peptide	N3/-DBCO	MCF7 and MDA-MB-231 cells; in vivo breast model	[38]
Leukocytes	Anti-PD-1 antibody	-N3/-DBCO	In vivo melanoma and breast models	[39]
CAR-T cells	-N3	-N3/-BCN	Raji cells; in vivo lymphoma model	[40]

RBC: red blood cell; NK: natural killer; MSC: mesenchymal stem cell; MC: macrophages.

### 2.2. Loading Strategies

Strategies for loading drugs onto cells for use as drug delivery systems involve two main approaches: intracellular loading and surface loading. Drugs may be incorporated either directly or conjugated to nanocarriers, such as nanoparticles, liposomes, or other nanostructured materials. The following sections summarize some commonly used methods.

#### 2.2.1. Intracellular Encapsulation

A frequently employed approach for loading therapeutics is their encapsulation within whole cells, either as free drugs or conjugated to NPs or other nanoformulations. Several methods can introduce compounds into the cytoplasm of living cells, including hypotonic loading, electroporation, and phagocytosis [7].

Hypotonic loading is widely applied as it induces minimal cellular alterations while providing high encapsulation efficiency compared to alternative methods. This approach encompasses various strategies, including hypotonic swelling, dilution, hemolysis, and dialysis [41], all of which rely on the formation of transient pores in a hypotonic environment. These holes allow soluble drugs to enter cells, driven by the concentration gradient, and subsequent restoration of isotonic conditions leads to pore closure, trapping the compounds inside. This method has been used to accommodate a broad range of agents, including small molecules, peptides, proteins, nucleic acids, and NPs [26,42,43,44,45,46].

Electroporation employs brief electrical pulses to transiently disrupt the cell membrane, forming temporary pores that enable cargo uptake. Once the pulse ends, the membrane reseals, entrapping the molecules. Pore size and membrane permeabilization can be finely tuned by adjusting the electrical parameters, allowing precise control over loading efficiency and cell recovery. While often applied to extracellular vesicles such as exosomes [47,48], electroporation is also suitable for live cells [49] and offers a fast, reproducible means of cargo incorporation.

Phagocytosis is particularly suited to cells with innate phagocytic activity. Studies report that therapeutic molecules or nanostructures can be internalized in this manner by immune cells [28,50,51,52] and platelets [53]. The procedure is quite straightforward, typically requiring only incubation of the cells with the cargo for a few hours. These cells are typically capable of englobing particles of various shapes and sizes, a feature that enhances their versatility [54,55].

One major advantage of internal drug encapsulation is the potential to achieve high cargo loading. However, excessive accumulation of cytotoxic agents, such as anticancer drugs, may compromise cell viability. To mitigate this, drugs can be encapsulated within protective structures that limit direct interaction with the intracellular environment. For example, coating doxorubicin with temperature-sensitive polar lipids was shown to lower toxicity in macrophage-based drug carriers [56]. Similarly, silica nanocapsules [57] and liposomes [58] have been employed to address the same challenge. Nevertheless, intracellular loading can result in invasive conditions, as the transient pore formation required for drug incorporation can compromise membrane integrity and trigger rapid elimination.

#### 2.2.2. Non-Covalent Surface Binding

Therapeutic agents can be anchored to the cell membrane through covalent or non-covalent bonds, with the latter often mediated by weak interactions such as hydrogen bonding or van der Waals forces. Non-covalent strategies may rely on non-specific adsorption, or on specific receptor–ligand interactions. Again, we can use as a notable example the red blood cells hitchhiking (RBC-hitchhiking), by which drug-loaded nanocarriers passively adsorb onto erythrocytes for targeted delivery to organs [59] a range of nanocarriers, including PLGA (Polylactic-co-glycolic acid) and polystyrene NPs, liposomes, and nanogels. This strategy is used to enhance pulmonary delivery while reducing hepatic uptake, with the lung-to-liver ratio varying according to the nanocarrier type [60]. In fact, owing to the distinctive biophysical properties of RBCs and their natural interactions with the pulmonary circulation, the lungs constitute a key target for RBC-based hitchhiking strategies [61]. NP release in the lungs is thought to occur as RBCs deform within narrow pulmonary capillaries, where shear forces promote transfer of adsorbed NPs to resident endothelial cells [59,61]. Preclinical studies have shown that chemotherapeutic-loaded NPs delivered by this method achieve more than a 15-fold increase in pulmonary accumulation compared to free NPs [62,63], with up to 40% of the injected dose localizing in the lungs [60,62]. Beyond the lungs, RBC-hitchhiking has also enabled delivery to challenging organs, such as the brain, where it allowed up to a tenfold enhancement in drug accumulation relative to other nanomedicine approaches [60].

As noted, endogenous cell-surface molecules can be exploited to establish non-covalent interactions. In particular, protein–protein interaction, such as antigen–antibody or receptor–ligand binding, has been widely used to anchor therapeutics to cellular membranes [20,64,65]. Notably, a recent approach exploits antigen–antibody interactions to exploit circulating RBCs for lung-targeted delivery, eliminating the need for ex vivo manipulation by targeting RBCs directly in the bloodstream [9]. In this strategy, liposomal carriers are functionalized with two antibodies: one directed against RBCs and another against pulmonary endothelial cells, forming the Dual Affinity to RBCs and Target cells (DART) platform. Optimization of this system enhances drug delivery and targeting precision while enabling RBCs to re-enter circulation after payload release [9].

#### 2.2.3. Covalent Surface Binding

Covalent attachment of drugs or nanocarriers to the cell surface follows the same principles and carries the same potential risks of plasma membrane alteration as those described for active targeting strategies. An important consideration, however, concerns the amount of drug that can be loaded onto the surface, regardless of whether covalent or non-covalent interactions are employed. Because the membrane is essential for critical cellular functions, surface cargo density must be carefully restricted to preserve its integrity. Several studies reported methods for covalent loading of therapeutics onto the cell surface by reaction with naturally occurring functional groups, such as amines, thiols, and hydroxyls [66]. Even in this context, the use of artificial, non-native functional groups for bio-orthogonal reactions offers a promising alternative for achieving stable and selective conjugation of therapeutics to biological membranes, as supported by a growing body of literature [17,67,68,69,70,71].

### 2.3. Drug Release

A fundamental aspect of a DDS is its release mechanism, which critically determines therapeutic efficacy and overall performance. Precise regulation of release is essential to minimize adverse effects and maximize treatment outcomes. Multiple factors influence drug release from cellular carriers and must therefore be carefully considered during design. For instance, small hydrophobic molecules may diffuse too rapidly across the carrier membrane, leading to premature leakage before reaching the tumor site. In addition, cells possess cytoprotective mechanisms, such as the P-glycoprotein efflux pump and exocytosis, that can actively expel drugs, further contributing to unwanted release [72]. These challenges highlight the importance of optimizing both drug retention and release kinetics.

**Figure 2:**
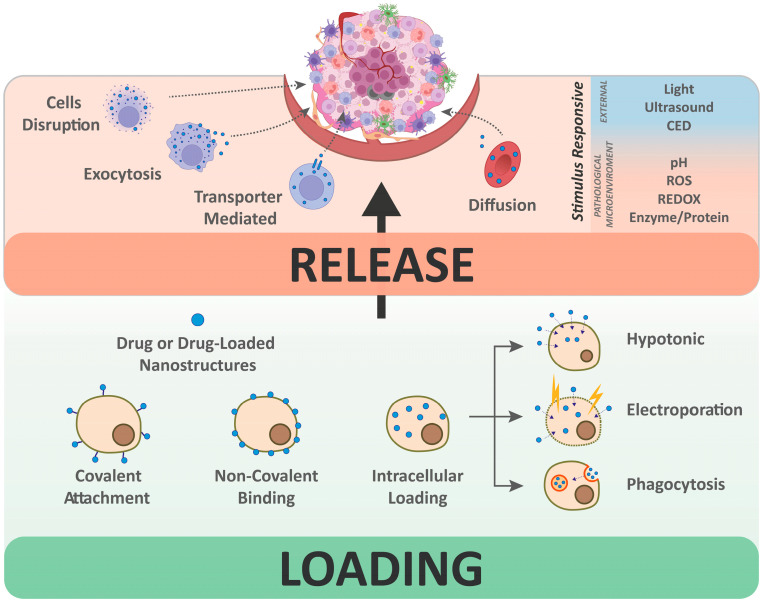
Drug loading and release strategies in cell-based drug delivery systems. Common drug-loading strategies are illustrated, including cell-surface decoration via covalent or non-covalent conjugation and intracellular loading through phagocytosis or transient membrane permeabilization achieved by electroporation or hypotonic treatment. Drug-release mechanisms include cell disruption, exocytosis, passive diffusion, and transporter-mediated export, with many pathways being activated by physiological or pathological stimuli.

Strategies to limit premature leakage include the use of membrane-impermeable prodrugs that are converted intracellularly into permeable forms, or the encapsulation of drug-loaded nanostructures rather than free drugs (Figure 2).

Contemporary DDS designs increasingly focus on achieving precise spatiotemporal control over drug release to maximize efficacy and minimize off-target effects. Many systems are engineered to employ external stimuli, such as ultrasounds [73,74,75], light [12,76,77], or a magnetic field [78] to trigger drug discharge. For example, echogenic, ultrasound-responsive microbubbles have been employed to disrupt macrophage membranes, inducing release of doxorubicin-loaded polymer vesicles upon focused ultrasound exposure [73]. Similarly, platelet-encapsulating porphyrin-based nanoparticles generated reactive oxygen species (ROS) under ultrasound stimulation, triggering platelet degranulation and release of the amino-acid transporter inhibitor α-Methyl-DL-Tryptophan [12]. Light-triggered strategies include the use of citric acid-coated photothermal superparamagnetic NPs loaded into macrophages to induce doxorubicin release from thermosensitive liposomes upon near-infrared (NIR) irradiation [12]. In a related approach, a porphyrin-derived photosensitizer attached to the RBC surfaces triggered membrane disruption and controlled drug release under mild laser irradiation [77]. Furthermore, magnetic field-responsive systems exploited macrophages co-loaded with drug-containing NPs and SPIONs. Exposure to an alternating magnetic field generates heat from SPIONs, inducing macrophage death and subsequent drug release at the target site [78].

More recently, attention has shifted toward systems responsive to endogenous cues intrinsic to pathological tissues, thereby confining therapeutic release specifically to the disease site. Tumor microenvironment, in particular, exhibits hallmarks such as aberrant pH, redox imbalance, elevated ROS levels, and the distinctive expression of specific proteins and enzymes that can be harnessed for controlled release [79]. Numerous cell-based carriers have been engineered to respond to pH changes [69,74,80,81], redox potential [67,82,83], ROS levels [84], and protein or enzymatic activity [85,86]. For instance, platelets loaded with metal–organic framework nanoparticles encapsulating α-Methyl-DL-Tryptophan (α-MT) and coated with a MnO_2_ shell prevented premature release, while selectively releasing cargo in response to the acidic pH and elevated glutathione levels of the tumor microenvironment [74]. Similarly, regulatory T cells functionalized with pH-responsive liposomes remained stable at physiological pH but destabilized in the acidic tumor microenvironment, triggering local release of immunomodulatory cargo, promoting dendritic cell maturation, checkpoint blockade, and effector T cell infiltration, thereby reshaping the tumor immune milieu while minimizing off-target effects [81].

Redox-responsive systems are designed, including, for example, CAR-T cells conjugated with disulfide-crosslinked, IL-12–loaded HSA nanoparticles. Upon antigen-triggered activation, increased cell-surface thiols cleaved the disulfide linkers, enabling localized, activation-dependent IL-12 release [67]. In another approach, stem cells carrying 5-ethynyl-2′-deoxycytidine (EdC)-loaded nanogels linked via disulfides are able to selectively release their cargo in the glutathione-rich tumor microenvironment [82].

ROS-induced release was achieved by loading the mitochondrial inhibitor Gboxin into ROS-sensitive polymeric nanoparticles coated with a cancer–mitochondria hybrid membrane. High ROS levels in tumor mitochondria cleaved the polymer linkers in the polymer, triggering site-specific drug liberation [84].

Protease-mediated release has also been exploited. Monocytes loaded with mertansine-bearing nanoparticles linked via protease-sensitive peptides released the drug locally upon differentiation into macrophages at metastatic lung sites, where tumor-associated protease legumain cleaved the linkers [85]. A similar strategy employed fibroblast activation protein protease (FAP)-sensitive phospholipid–DM4 prodrug conjugates anchored to M1 macrophage membranes; accumulation in metastatic lesions triggered FAP-mediated cleavage, releasing the potent cytotoxic agent DM4 specifically at the tumor site [86].

In here, we have outlined both classical and innovative technical advances underpinning emerging therapeutic strategies that integrate cell-based carriers responsive to endogenous tumor cues. Collectively, these approaches hold substantial promise for enabling highly selective and spatiotemporally controlled drug delivery, thereby enhancing therapeutic efficacy while minimizing off-target effects. Equally critical, however, is the careful selection of cell types and their specific biological features to ensure optimal delivery performance. This consideration is particularly crucial for so-called “hard-to-treat” malignancies, such as intracranial tumors including glioblastoma, where therapeutic intervention is severely hindered by the presence of the blood–brain barrier.

## 3. Cells Crossing Barriers on the Path to the Brain

### 3.1. The Blood–Brain Barrier

Paving the surface of the vasculature in the brain, the BBB is a highly selective and tightly regulated interface that protects the brain by restricting the entry of most circulating substances. However, this protective function also presents a major challenge for delivering therapeutic agents, as it restricts the drug’s access to its intended targets. Brain capillary endothelial cells, together with astrocytes and pericytes, preserve and regulate exchanges between blood and brain parenchyma, maintaining homeostasis. Endothelial cells are connected by tight junctions lacking fenestrations, and exhibit low pinocytic activity, limiting passive transport to small molecules such as oxygen and lipid-soluble compounds. However, crossing the BBB may occur via both active and passive mechanisms [87], and transient disruptions, such as osmotic shock or damage, can temporarily allow uncontrolled passive transport of drugs.

Some nutrients, such as glucose, cross the BBB via specific carriers (e.g., GLUT1) that mediate facilitated diffusion along a concentration gradient without energy consumption. In contrast, most essential molecules—including many therapeutic agents—rely on active transport systems. These include carrier-mediated transport for amino acids, receptor-mediated transcytosis for larger proteins like transferrin and insulin, and adsorptive-mediated transcytosis for cationic proteins and peptides. Receptors such as the transferrin receptor and epidermal growth factor receptor (EGFR) enable the selective uptake of ligands via vesicular transport. Pericytes and astrocytes support vascular integrity and modulate transporter expression [87]. Thus, while BBB restricts general molecular entry, it provides specialized pathways for controlled and selective transport of critical substances.

Another biological membrane, the cerebral spinal fluid–blood barrier (CSFB), also regulates the movement of substances from and to the brain [88], though this route has not been effectively exploited for cell mediated drug delivery, maybe because accessing to the brain via the CSFB, which can be achieved through intraventricular or lumbar injection, results in largely non-specific delivery [89].

### 3.2. Glioblastoma

Glioblastoma (GBM) is the most aggressive form of adult-type diffuse glioma, a tumor originating from glial cells in the central nervous system (CNS). According to the World Health Organization (WHO) classification of CNS tumors [90], glioblastoma is an isocitrate dehydrogenase (IDH)-wildtype CNS WHO grade 4 tumor, characterized by the absence of mutations in IDH genes, the presence of microvascular proliferation and/or necrosis, and at least one predictive molecular alteration, including EGFR amplification and telomerase promoter (TERTp) mutations [91], as well as chromosomal changes such as gain of chromosome 7 and loss of chromosome 10 [92].

Despite significant scientific efforts and technological advancements, glioblastoma remains incurable. Many innovative therapeutic strategies ultimately fail, primarily due to the tumor’s heterogeneous nature, the development of drug resistance, and, most critically, the presence of the BBB, which restricts drug access to the tumor. In fact, less than 20% of temozolomide (TMZ)—the standard oral treatment used in the Stupp protocol for glioblastoma—crosses the BBB, and even less TMZ reaches the tumor site [93]. Consequently, high doses of repeated TMZ cycles are required, leading to severe side effects for patients.

### 3.3. Cell-Mediated Delivery to the Brain

To overcome these limitations, strategies for effectively targeting glioblastoma beyond the BBB include systemic approaches (intravenous, intraperitoneal, or oral administration), intratumoral administration (e.g., convection-enhanced delivery, intratumoral injection), and locoregional treatments employing various drugs and drug delivery systems [94]. Several of these strategies utilize cell-based delivery systems. These biological carriers can effectively reach the brain by exploiting innate mechanisms such as transcytosis or inflammation-mediated BBB disruption, responding to chemotactic signals to home diseased tissues [95].

Such strategies offer hope for a more effective glioblastoma treatment and for potential applications in other neurological conditions, including neurodegenerative diseases and stroke. Here, we highlight the most relevant features of each cell type and discuss how they have been exploited for the recent advances in brain-targeted drug delivery, achieved through bio-manipulation and bioengineering (Table 2).

#### 3.3.1. RBCs

Human RBCs are the predominant cellular component of the bloodstream. Mature RBCs are enucleated and lack other organelles, adopting a distinctive biconcave shape that enables them to squeeze through narrow capillaries, while providing a large intracellular volume and a highly deformable membrane, features advantageous for drug encapsulation and delivery. Their long circulating lifespan of about 120 days is particularly advantageous for prolonged therapeutic release [7,153]. Despite lacking organelles, RBCs retain metabolic enzymes capable of converting prodrugs into active, membrane-permeable forms, thereby minimizing premature leakage, prolonging drug half-life, and supporting sustained release [154].

Drugs are typically loaded into RBCs by hypotonic swelling, although alternative strategies, including electroporation [155], liposome fusion [156], and the use of cell-penetrating peptides [157], have also been reported. In addition, RBC surface loading, particularly through the “hitchhiking approach”, has been used to transport a wide range of therapeutics, including anti-inflammatory, antiviral, and anticancer agents, as well as enzymes, antibodies, antigens, and nucleic acids, demonstrating their remarkable versatility [158].

Drug release from RBC carriers occurs primarily via passive diffusion of permeable drugs, enabling prolonged drug exposure. Because RBCs lack lysosomes and exocytotic capability, large complexes, polar drugs, and proteins cannot exit by these routes and are instead released upon carrier disruption [159]. For certain compounds, such as doxorubicin, active transporter-mediated release may also occur due to efflux transporters on the RBC membrane [160].

The safety and efficacy of RBC-based carriers have been demonstrated in multiple clinical trials, with several therapeutic products based on this technology advancing into late-stage development. One prominent example is EryDex, in which autologous RBCs are loaded with dexamethasone sodium phosphate for the treatment of the genetic disorder ataxia-telangiectasia [161].

An RBCs-based delivery platform named Erythro-Magneto-Hemagglutinin-Virosomes (EMHVs), surface-decorated with anchoring proteins and enriched with magnetic NPs, can be concentrated in selected body regions using an external magnetic field. The concentrated EMHVs can easily fuse with the target cells to deliver chemotherapeutics [162] or immunotherapeutic agents [26]. Notably, the accumulation of systemically administered EMHVs into the brain under an external magnetic field was predicted by the design of a magnetic-field-generating helmet for the treatment of cerebral tumors [137].

#### 3.3.2. Mesenchymal Cells

Mesenchymal stem cells (MSCs) are multipotent stromal cells with robust self-renewal capacity and the ability to differentiate into multiple mesodermal lineages. They are present in a wide range of tissues, including bone marrow, umbilical cord, peripheral blood, adipose tissue, skin, and dental pulp, and are characterized by low immunogenicity [7,163]. Umbilical cord-derived mesenchymal stem cells are most employed, either alone or in combination with Multilineage Differentiating Stress Enduring (MUSE) cells.

MSCs exhibit pronounced tumor tropism due to their natural ability to migrate toward sites of injury in response to chemotactic gradients of inflammatory cytokines. They possess a strong tumor-penetration capacity, enabling them to reach internal, hypoxic, and poorly vascularized regions of tumor tissues. Several MSC-based therapies for degenerative diseases have already received approval, while others are under clinical investigation, collectively supporting their favorable safety profile [163].

As MSCs display moderate to strong resistance to a broad range of chemotherapeutic agents, including cisplatin, vincristine, gemcitabine, doxorubicin, and paclitaxel, this intrinsic resistance allows them to serve as carriers for both free drugs and drug-loaded NPs and release them at primary and metastatic tumor sites [164]. Most anticancer drugs are readily absorbed from the surrounding medium, enabling straightforward loading strategies. For example, in vitro isolated MSCs readily internalize gemcitabine, doxorubicin, cisplatin, and paclitaxel upon sole exposure, whereas uptake of pemetrexed is limited. Drug loading inside these cells can be achieved by simple diffusion, endocytosis, or hCNT1 and hENT1 transporters, depending on the chemical-physical nature of the molecules [164]. Alternatively, therapeutic compounds can be conjugated to the MSC membrane using classical covalent or non-covalent surface binding strategies [71,165]. Notably, drug release by MSCs primarily often occurs via extracellular vesicles, which serve as a major mode of communication between MSCs and tumor cells [164].

Despite their advantages, MSCs exhibit characteristics that require careful consideration. Beyond serving as drug carriers, they can significantly influence tumor progression through their immunoregulatory capabilities on the tumor microenvironment. In fact, they can display immunosuppressive properties, enabling tumor cells to evade immune surveillance and promoting drug resistance, angiogenesis, tumor growth, and metastasis. Conversely, under certain conditions, MSCs can act in a tumor-suppressive manner by inhibiting pro-survival signaling pathways, suppressing angiogenesis, inducing cell cycle arrest and apoptosis, and enhancing inflammation and immune cell infiltration [166]. To leverage their tumor-suppressive potential, MSCs can be engineered to express or deliver anti-proliferative, pro-apoptotic, or anti-angiogenic agents, thereby promoting antitumor function [166]. Mesenchymal cells are frequently used in cellular therapies for neurological diseases [167], exploiting their natural homing ability to reach sites of cerebral damage and bypass the protective barriers of the BBB and the blood–tumor barrier (BTB) [168]. To minimize unintended side effects in peripheral organs, mesenchymal cells have been engineered to carry antiproliferative miRNAs [99], CRISPR-Cas9-edited immune modulators, or T-cell activator-mediated tumor-killing agents [106,107] and delivered beyond the BBB via internal carotid artery infusion, intracranial implantation, or magnetic field–mediated local concentration [100].

In some cases, adipose-derived stem cells have been used to reach certain brain regions through intranasal administration. Intranasal delivery is generally considered a direct, safe, and rapid route to the brain for various therapies. While it holds promise for delivering anti-inflammatory treatments that do not require precise regional or molecular targeting, this route is less suitable for glioblastoma, which often occurs in cerebral regions that are distant from the olfactory system, limiting local drug bioavailability [169].

#### 3.3.3. Adoptive Cell Transfer (ACT)

Adoptive cell transfer (ACT) therapy emerges as a potent strategy that involves ex vivo manipulation of patient-derived immune cells, such as macrophages, tumor-infiltrating lymphocytes, neutrophils, and natural killer cells, followed by their reinfusion into the host to enhance antitumor immunity. By reprogramming the TME toward an “immune-hot” and tumoricidal state, ACT counteracts the immunosuppressive and anti-infiltrating characteristics of the tumor niche. In glioblastoma, the TME comprises cancer stem cells, GASCs, endothelial cells forming the vasculature and the BBB, as well as various immune cells, including resident microglia, tumor-associated macrophages, and lymphocytes. Within this intricate microcosm—much like a forge—each component contributes to tumor progression by providing trophic support, facilitating oxygen and cytokine exchange, promoting cellular infiltration, and establishing a protective niche that shields the tumor from host immune surveillance.

ACT can exploit the homing ability of tumor-associated macrophages to counteract the immunosuppressive glioblastoma microenvironment. Macrophages, derived from circulating monocytes, reside in virtually all tissues, where they eliminate pathogens and regulate immunity, homeostasis, and tissue repair. Their remarkable plasticity allows reversible polarization into M1 or M2 phenotypes: M1 macrophages mediate pathogen clearance and anticancer responses via pro-inflammatory cytokines production, whereas M2 macrophages support tissue repair and often promote tumor progression through anti-inflammatory cytokines, extracellular matrix remodeling, and angiogenesis [170,171]. Their innate ability to home inflammed tissues, including tumors, combined with the capacity to cross biological barriers and infiltrate hypoxic, poorly vascularized regions, makes macrophages attractive candidates for delivering therapeutics, even to challenging sites such as the brain [50,172,173].

Because macrophages can phagocytose structures of diverse shapes and sizes [54,55], this property is frequently exploited for intracellular loading of therapeutics. In addition, surface “backpacking” strategies are also employed [75,174]. To further minimize cellular damage, macrophages are often engineered to transport drug-loaded liposomes or NPs instead of free cytotoxic agents [50,56,57,175].

M1 macrophages are increasingly favored as drug carriers over unpolarized macrophages because of their superior phagocytic capacity and intrinsic antitumor activity, which enables both efficient therapeutic uptake and direct tumor inhibition [176]. Tumor-associated macrophages (TAMs), which can comprise up to 50% of the tumor mass, are typically polarized to an M2 phenotype that supports cancer progression. However, their plasticity allows reprogramming toward an M1 phenotype, thereby turning them into potential antitumor effectors. For example, some researchers demonstrated that M1 macrophages can deliver NPs that re-educate resident TAMs toward the M1 phenotype, thereby boosting their intrinsic antitumor activity [51]. These findings highlight the necessity of monitoring macrophage phenotype during therapeutic delivery and of employing strategies that stabilize M1 polarization within the immunosuppressive tumor microenvironment [174,176].

Macrophages loaded with drug–NP complexes have been shown to discharge their payloads at the tumor site via exocytosis [57], a process that can be further accelerated by tumor-associated inflammation and microenvironmental cues [50]. These complexes may be excreted intact or as dissociated components, suggesting that disassembly may occur within the cellular carrier. Lysosomes are likely central to this process, as their acidic environment and hydrolase activity can facilitate drug detachment from NPs [177].

Although macrophage-based drug delivery remains at the preclinical stage, their unique biological properties and the advancement of macrophage-focused clinical trials provide a strong rationale for translating these cells into genuine drug-delivery platforms.

Neutrophils are the most abundant class of leukocytes and key effectors of the innate immune system, rapidly responding to sites of infection and inflammation. Their natural ability to infiltrate tumor tissues, guided by chemotactic signals from the tumor microenvironment, along with the capacity to bypass the BBB, makes them attractive candidates for targeted anticancer drug delivery. Therapeutic cargo can be introduced into neutrophils through several strategies, most prominently phagocytosis and endocytosis, enabling efficient internalization of drug-loaded NPs or liposomes during ex vivo incubation and subsequent transport to tumor sites [178]. Smaller NPs or chemically functionalized carriers can also be internalized via receptor-mediated pathways, with receptor-specific surface modifications further enhancing uptake efficiency [178]. Beyond ex vivo loading, several studies have harnessed the ability of circulating neutrophils to directly sequester therapeutic NPs in vivo, although the efficiency of this process is highly dependent on the physicochemical properties of the NPs [179]. In addition, therapeutic cargos can be anchored directly to the neutrophil membrane using established conjugation strategies [178].

Drug release from neutrophils is largely driven by their responses to the tumor microenvironment. Upon entering tumor tissue, neutrophils are activated by inflammatory signals, cytokines, and reactive oxygen species, which can trigger degranulation, oxidative bursts, or NETosis, a process in which neutrophils release extracellular traps (NETs), resulting in the discharge of internalized therapeutic cargo [178]. Neutrophil apoptosis or secondary necrosis also contributes to payload release, further promoting localized delivery. On the other hand, the short half-life of circulating neutrophils (≈7 h) limits their ex vivo manipulation and clinical translation, as their rapid clearance narrows the time window for effective drug delivery after reinfusion [178]. Moreover, using the neutrophil system in cancer therapy requires careful consideration, as they can exert dual and opposing roles within the tumor microenvironment. On one hand, they can enhance antitumor immunity by releasing cytotoxic mediators and recruiting additional immune effector cells. Conversely, they may promote tumor progression by secreting pro-inflammatory cytokines, stimulating angiogenesis, and suppressing immune surveillance [180]. To address this challenge, current research focuses on strategies that suppress the immunosuppressive activities of neutrophils while shifting their functional state to favor tumor suppression [180].

Recently, in an orthotopic mouse model of glioblastoma, neutrophils were decorated with immunostimulant Cyto-Adhesive Micro-Patches (CAMPs) and used in combination with checkpoint inhibitors to induce activation of the immune system [146], resulting in neutrophil accumulation at tumor sites. Similarly, neutrophils can be loaded with TMZ-containing cholesterol nanoparticles, exposing T7, a cell membrane-penetrating peptide, to optimize TMZ delivery [145]. Applying a different strategy, neutrophils were loaded with a doxorubicin-enriched polymeric photosensitizer to increase the local concentration of antiproliferative ROS in glioblastoma models [144].

T lymphocytes, key components of the adaptive immune system, mature in the thymus and enter the circulation as naïve T cells. Upon encountering their cognate antigen, they undergo a multistep activation process, resulting in proliferation and the initiation of immune responses. Tumor-infiltrating lymphocytes (TIL) represent a selected population of these reactive immune cells with antigen specificity that can be isolated from patient tumors and, in some cases, expanded to create a personalized reservoir of autologous cells. For example, they have been “backpacked” with liposomes carrying IL-2, PD-L1, and imiquimod to promote dendritic cell maturation, inhibit PD-1/PD-L1 signaling, and enhance CD8^+^ T-cell infiltration into tumors [81]. They have also been loaded with nanogels releasing IL-15 superagonist to boost T-cell expansion and function [83] and with nanoparticles carrying a topoisomerase I inhibitor for localized chemotherapy [181].

T cell-based drug delivery systems can release their cargo through multiple mechanisms, including passive diffusion [181] or stimuli-responsive mechanisms triggered by tumor-specific cues [81].

The difference in immune cell composition in the suppressive tumor microenvironment, which includes type 1 helper T cells and activated B cells, as well as tumor-reactive T cells, contributes to the cell heterogeneity, which is a signature of GBM [182]. In the attempt to favor immunoreactivity, autologous lymphocytes expressing early activation marker CD69 and CD137 can be reinfused into patients to enhance antitumor activity [182]. TIL genomic characterization revealed the potential of TIL therapy against the heterogeneity of the native tumor and suggests a future development of personalized therapy, like what is already seen in immune-permissive solid tumors.

#### 3.3.4. CAR-T Cells Crossing the BBB

In recent years, T-cell-based cancer therapies have gained significant momentum, most notably with the development of CAR-T cells. This form of immunotherapy involves genetically engineering patients’ T cells to express a chimeric antigen receptor (CAR) that recognizes specific proteins on cancer cells, enabling targeted elimination of malignant cells. CAR-T therapy has shown remarkable success in certain hematological malignancies; however, challenges persist in solid tumors, where the immunosuppressive features of the TME limit T-cell infiltration, for example, in glioblastoma.

To address this issue, several studies have demonstrated that CAR-T cells can also be engineered to deliver compounds using the “backpack” strategy, which helps overcome the hostile tumor environment and enhance T-cell infiltration and activity. In this context, T cells assume a dual function: serving as vehicles for targeted delivery while maintaining their primary role as cell therapy agents [183]. Alternatively, CAR-T cells can be genetically programmed to express inducible cytokines, enhancing therapeutic efficacy while modulating the TME [184]. In fact, biotechnological advancements have progressively enhanced the specificity and efficiency of CAR-T cell vectors by refining T lymphocyte signaling. First-generation CAR-T cells improved extracellular antigen-specific recognition, while later generations (second to fourth) increased intracellular chimeric complexity to enable secretion of co-stimulatory molecules, such as CD28 and 4-1BB [185], and cytokines such as IL-12 [186], amplifying the immune response and sustaining T-cell survival [187,188]. Fifth-generation CAR-T cells incorporate cytokine receptor-derived signaling domains that engage transcription factors such as STAT3 to further activate cytokine cascades [189].

Since the early successful attempts to use lymphocytes for delivering polymeric nanoparticles across the BBB into the immune-cold tumor environment [190], CAR-T therapy has become the focus of numerous clinical trials aimed at treating intracranial tumors with cell-based immunotherapy (clinicaltrials.gov) (Table 3). Over one hundred articles reviewed the use of CAR-T therapy in glioblastoma, highlighting its tremendous therapeutic potential and technological versatility. Most recent clinical trials employ adoptive therapy with autologous CD4^+^ and CD8^+^ T cells transduced to express CARs directed against immunomodulatory or tumor-associated proteins such as B7-H3 (CD276), interleukin-13 receptors (IL-13Rα2), epidermal growth factor receptor variant III (EGFRvIII), either individually or in combination (NCT05168423; NCT06186401; NCT07193628; NCT0720924; NCT07209241). These engineered CAR-T cells simultaneously recognize and destroy tumor cells, thereby enhancing the precision and efficacy of the immune response. CAR-T therapy also incorporates co-stimulatory domains, such as 4-1BB, to enhance T-cell activation and persistence (NCT02208362; NCT05063682; NCT04003649). The use of CAR-T cells also enables multi-targeted and multi-step therapeutic strategies. For example, Tris-CAR-T cells have been engineered to recognize multiple antigens, such as CD44 and CD133, which are inversely expressed on distinct tumor subpopulations. This dual targeting approach aims at preventing immune escape, which is particularly common in glioblastoma stem cells. Moreover, the introduction of a truncated form of IL-7 receptor within the intracellular portion of the chimeric antigen receptor can delay CAR-T cell exhaustion and prolong tumor suppression (NCT05577091) [191]. In another approach, CRISPR-Cas9-mediated knockout of transforming growth factor beta receptor 2 in T cells was introduced in IL-13Rα2-directed CAR-T cells (NCT06815029) to silence TGF-β signaling and counteract tumor-mediated immunosuppression. Moreover, Epidermal Growth Factor Receptor (EGFR)-targeted CAR-T can be engineered to deliver the programmed cell death protein 1 (PD-1) inhibitor pembrolizumab directly to the tumor site (NCT03726515), thereby facilitating localized immune checkpoint blockade and enhancing antitumor immunity.

Most of the clinical trials are designed to evaluate the safety and feasibility of therapies administered locoregionally, in order to maximize efficacy while minimizing adverse effects. To this end, various delivery methods have been employed, including intratumoral or intracranial injections, Ommaya reservoir implantation, intracerebroventricular (ICV) injection, and convection-enhanced delivery (CED). Interestingly, this locoregional approach is somewhat counterintuitive, given that lymphocytes can naturally cross the BBB. Indeed, intravenous (IV) administration has been less commonly used. Nevertheless, recent proof-of-principle and preclinical studies with the use of CAR-T are designed to explore systemic delivery strategies to improve antigen specificity and immunogenicity (Table 4). Ganglioside GD2, a membrane molecule associated with high malignancy, has been used together with the prostate-specific membrane antigen (PSMA) [192] in a fourth-generation CAR-T-based therapy of refractory glioma. Moreover, to sensitize medulloblastomas to immunotherapy, GD2-CAR-T can be used in combination with FLASH therapy, a state-of-the-art radiotherapy characterized by a very high dose of radiation in milliseconds to facilitate TME lymphocytes infiltration [193]. Similarly, combinatorial strategies have been developed using anti-CD87 and CD3 bispecific T-cell engagers (CD87/CD3 BITEs) in conjunction with IL-12-expressing CAR-T cells, demonstrating antigen-specific cytotoxicity in nonfunctioning pituitary adenomas [194]. Innovative CAR designs are created to target different molecular epitopes to overcome tumor glioblastoma heterogeneity, such as the Prostaglandin F2 receptor negative regulator (PTGFRN) [195] or employing streptavidin-based systems to enhance interactions with biotinylated antigens [195].

Despite their continued evolution, however, CAR-T cells still face key challenges, including limited infiltration into the tumor microenvironments and off-target immune toxicities reported in preclinical studies [196]. A recent report, in fact, denounced a potential toxicity of CAR-T therapy in hematologic malignancies, also described as immune effector cell-associated neurotoxicity (ICANS), mostly associate with impairment of memory function [196,197] sustained by increased cerebrospinal fluid cytokine levels and disruption of the blood–brain barrier. Together with an increase in circulating cytokines such as TNF-α and IFN-γ that in turn activate secretion by monocytes and macrophages of additional cytokines, including IL-1, IL-6, and nitric oxide synthase (iNOS), CAR-Ts might sustain hyperinflammatory reactions causing damage to peripheral organs such as live and kidney. In GBM tumors, inflammation-associated neurotoxicity (TIAN) may impinge on the mechanistic aspects of the neuronal activity giving rise to neurological deficit (TIAN1). On the other hand, neurotoxicity may also cause tissue hyperexcitability (TIAN2) [198]. However, given the transient, subtle nature of these symptoms, it is debatable whether the risk of neurotoxicity associated with CAR-T therapy might prevent the use of an effective cure for glioblastoma.

**Table 4:** Recent development of CAR-T-based brain-targeted drug delivery systems.

Modification	Delivery	Agent/Drug	Treatment	Disease	Refs.	Year
αβ T cells engineered with a high-affinity γ9δ2 T-cell receptor (TEGs) recognizing virally infected cells via BTN2A1 and BTN3A	In vitro	OVs and Vγ9Vδ2 TCR	OV therapy and increased immunotherapy	Pediatric Diffuse Midline Gliomas (DMGs)	[199]	2025
Prostaglandin F2 receptor negative regulator (PTGFRN)-targeting 5E17-CAR-T cells	IC	5E17-CAR-T	Immunotherapy	Glioblastoma	[200]	2025
NKG2D CAR-T cells combined with sodium valproate (VPA)	IV	NKG2D CAR-T + VPA	Antitumoral activity	Glioblastoma	[201]	2025
Gamma delta (γδ) T cells	In vitro	Gamma Delta (γδ)T cells	Immune infiltration	Medulloblastoma	[202]	2025
Fourth-generation combined PSMA- and GD2-targeted chimeric antigen receptor (CAR)-T cells	IV	PSMA/GD2 CAR-T cell	Immunetherapy	Refractory Glioma	[192]	2025
FLASH therapy combined with GD2 CAR-T cell immunotherapy	IC	GD2 CAR-T	Sensitization by radiation and reverse immunosuppression	Medulloblastoma	[193]	2025
CD70-specific CAR-T cells transduced with two third-generation oncolytic adenoviruses (OAds; E1B19K/E3-deleted, replication-selective): OAd-GFP (control) or OAd-IL15 (TS-2021)	IT	CAR-TOAd-GFP and CAR-TTS-2021	Viral oncolysis/immunotherapy	Glioblastoma	[203]	2025
CD44/CD133 dual-targeting CAR-T cells	IC	Tanζ-T28-Δ7R CAR-T cell	Antitumoral activity	Glioblastoma	[191]	2025
CAR-Vδ1 T cells targeting B7-H3 and IL-13Rα2	IT	CAR-Vδ1 T cell cocktail	Multi-step strategy for CAR-Vδ1 T cell cocktail therapy	Heterogeneous Glioblastoma	[195]	2025
CAR-T cells utilizing monomeric streptavidin-2 (mSA2)	IT	mSA2 CAR-T	Antiproliferative and heterogeneity targeting immunotherapy	Glioblastoma	[195]	2024
CAR-T cells targeting B7-H3	IC	B7-H3 CAR	Antitumor	Glioblastoma	[204]	2025
Bispecific T-cell engagers (BiTEs) and chimeric antigen receptor (CAR)-T cells	IC	anti-CD87 BiTE and CD87-specific CAR/IL-12 T	Increased immunogenicity and immunotherapy	Invasive nonfunctioning Pituitary Adenomas (iNFPAs)	[194]	2024
Antigen-sensitive B7-H3-targeting nanobody-based CAR-T cells	IV	B7-H3 nanoCAR-T	Tumor growth control	Glioblastoma (Xenograft)	[205]	2024
Chimeric antigen receptor (CAR)-T cells producing IL-7 and chemokine (C-C motif) ligand 19 (CCL19)	IV	IL 7 × 19 CAR-T	Antiproliferative	Glioblastoma/Pancreatic Cancer	[206]	2024
Chimeric antigen receptor (CAR)-T cell therapies targeting glioblastoma-associated antigens such as interleukin-13 receptor subunit alpha-2 (IL-13Rα2)	IV	IL-13Rα2/TGF-β bispecific CAR-T	Reduction of immunosuppression via TGF-β	Glioblastoma	[207]	2024
Chimeric antigen receptor (CAR)-modified T cells targeting GD2	IV/IC	GD2-CART	Tumor regression	H3K27M-Mutant Diffuse Midline Gliomas (DMGs)	[207]	2024

IC: intracranial injection; IV: intravenous injection; IT: intratumoral injection.

#### 3.3.5. Cell-Derived Drug Delivery Systems

Although this work focuses primarily on whole cells, it is also important to highlight certain cell-derived nanostructures due to their widespread use. For comprehensive discussions, we refer readers to recent reviews [208,209]. Cell membrane-coated nanoparticles and exosomes deserve special attention, as they offer highly effective platforms for tumor-targeted, cell-based drug delivery.

Synthetic NPs are widely explored as drug carriers due to their tunability and ease of chemical modification. However, poor biocompatibility and rapid clearance by the MPS limit their clinical use, with typically less than 1% of the administered dose reaching the target site [210]. To overcome these barriers, recent strategies have focused on cloaking drug-loaded NPs with cells or cell-derived membranes obtained by disrupting parent cells [211]. The resulting cell membrane-coated NPs, or biomimetic NPs, integrate the advantages of both nanomaterials and natural cell membranes, showing improved biocompatibility and extended circulation time due to enhanced immune evasion [212].

RBC membrane-coated NPs, commonly called nanoerythrosomes, benefit from their smaller size compared to whole RBCs, allowing them to extravasate more easily through leaky tumor vessels and passively accumulate in solid tumors via the enhanced permeability and retention (EPR) effect [212]. Nanoerythrosomes loaded with metformin have demonstrated the ability to bypass the BBB and have shown promising results in the in vitro treatment of glioblastoma [136]. In addition, coating nanoparticles with RBC membranes functionalized with targeting and cell-penetrating peptides enabled the systemic delivery of chemotherapeutics such as docetaxel [134] and temozolomide [213] to brain tumors.

Furthermore, by exploiting the inherent tropism of selected cell types or by functionalizing membranes with targeting ligands, it is possible to achieve more accurate delivery to pathological sites [214,215,216].

Moreover, numerous studies report the successful use of anti-tumor drug-loaded NPs coated with membranes from diverse sources, including RBCs [217,218], platelets [219,220], immune cells [211,221], stem cells [215,222], cancer cells [216,223], and even organelles [84]. An interesting aspect is the possibility of creating hybrid membranes assembled from multiple cellular origins. Because the protein composition of a plasma membrane dictates the biological behavior of the biomimetic system, hybrids retain the unique features and, thus, the advantages of all parent sources [214]. Notably, in antitumor therapy, hybrid membranes are often partly derived from tumor cells, to take advantage of their natural capacity of homotypic targeting [84,224,225,226,227].

Exosomes, extracellular vesicles secreted by both eukaryotic and prokaryotic cells, represent another naturally derived nanocarrier with complementary advantages [228]. Although their full functions are not yet completely understood, exosomes mediate paracrine-like cell-to-cell communication [229]. They carry a molecular “dowry” from their parent cells, including DNA, RNA, metabolites, lipids, and cytosolic or membrane-bound proteins [230], which can serve as biomarkers [231] or enable targeting of actionable sites within the tumors [232]. Exosomes can be engineered inside the cell of origin before extrusion (genetic/metabolic engineering) or after isolation (electroporation, sonication, incubation, chemical conjugation) to carry therapeutic molecules and can be functionalized with targeting ligands for selective delivery to specific tissues [208]. This makes them highly versatile for precision drug delivery.

Recently, exosomes have been widely employed as drug delivery carriers to the brain. They can be engineered to deliver anti-inflammatory [109,114,117,120] and neuroprotective treatments [110,111,115,116,122,127]. Some exosome-based therapies have been designed for high specificity, incorporating RNA-silencing nucleotides, such as siRNA targeting β-site precursor protein lyase-1, for the treatment of Alzheimer’s disease [111,118].

Therapeutic exosomes for glioblastoma are often delivered systemically via intravenous or intraperitoneal injection to exploit their ability to cross the BBB and transport agents such as anti-inflammatory [126], antiangiogenic [112], and monoclonal antibody therapies [113] to the tumor site. Examples include exosomes loaded with the proteasome inhibitor Celastrol [126], the immunosuppressants Rapamycin or Sirolimus in combination with chemotherapy [112], and the monoclonal antibody Bevacizumab [113]. Therapeutic efficacy can be enhanced by functionalizing exosome surfaces with epitopes, such as CpG nucleotides, to induce antitumor immune responses [108], or by inducing the expression of CD19, as demonstrated in targeted methotrexate delivery for CNS lymphoma [124]. Several studies have explored the potential use of exosomes or exosome-biomimetics [233] encapsulated within nanostructures or gel matrices to enable localized implantation. Recently, 3D-encapsulated delivery strategies have been applied primarily in cases of traumatic brain injury; however, the development of gel- or matrix-based, cell-mediated therapies for glioblastoma is now within reach [169].

Noteworthy, glioblastoma-derived exosomes have been described as an innovative, versatile, and specific tool to deliver therapeutic agents to the brain in glioblastoma [228]. Exosome production can occur locally, in proximity to the tumor mass, for example, by glioma-associated stem cells (GASCs). GASCs are non-tumorigenic but retain a mesenchymal, multipotent phenotype, providing diagnostic insights and reflecting the heterogeneity and severity of gliomas. To exert their tumor-supporting functions, these cells communicate with neighboring cells via exosome release, transferring numerous miRNAs that modulate tumor-related processes [234]. In vitro studies indicate that GASC-derived exosomes from low- or high-grade gliomas can differentially alter neuronal electrical properties, contributing to tumor-induced hyperexcitability and epileptic seizures [235,236].

## 4. Current Advances in Theranostic Cell-Based Delivery Systems in Oncology

**Figure 3:**
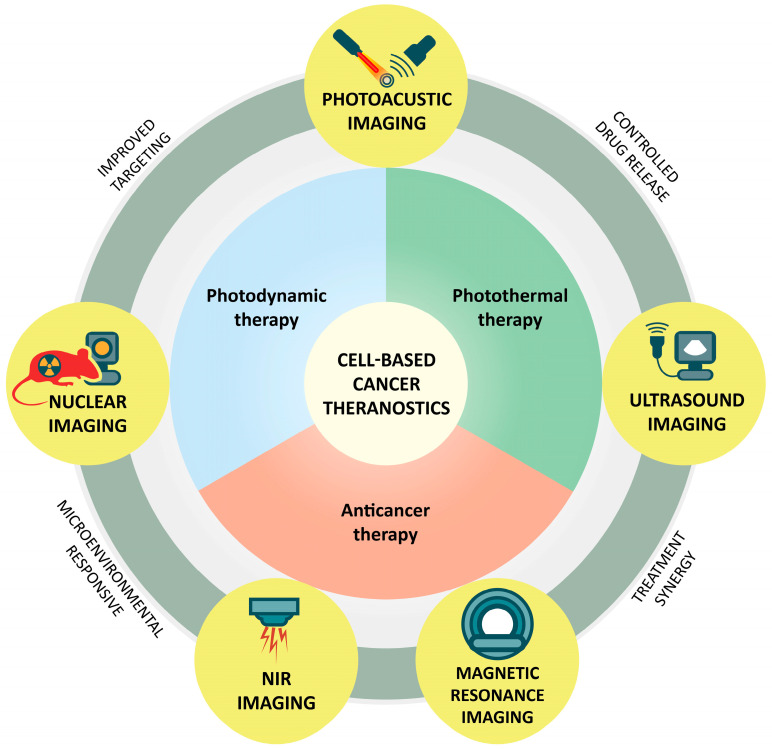
Cell-based cancer theranostics. Cutting-edge theranostic strategies can help improve precision medicine through targeted drug delivery and increased therapeutic efficacy during diagnosis.

Over the past few decades, the versatility of cellular systems has enabled their evolution from conventional drug delivery vehicles into multifunctional theranostic platforms, capable of carrying therapeutic payloads while providing imaging capabilities (Figure 3). These innovations offer major advances in oncology, allowing targeted therapy, improved diagnostic precision, and real-time, non-invasive monitoring of disease progression and treatment response. Theranostic carriers can be visualized by various imaging techniques, including Magnetic Resonance Imaging (MRI), Near Infrared (NIR) imaging, ultrasound (US), Photoacoustic Imaging (PAI), Positron Emission Tomography (PET), each with distinct advantages and limitations in tissue penetration, sensitivity, and spatial, contrast, and temporal resolution. Many of the cellular platforms, including RBCs, platelets, macrophages, neutrophils, natural killer cells, MSCs, and T cells used as carriers for anticancer drugs can also be enriched with different tracers that, coupled with advances in imaging, enable precise tracking of drug delivery [237].

RBC-based platforms have been extensively explored for theranostics. The FDA-approved fluorochrome indocyanine green (ICG) is a NIR-activatable dye that functions as a photosensitizer, enabling photodynamic therapy. Encapsulation of ICG within nanosized RBC carriers allowed fluorescence imaging of ovarian cancer cells in vitro, and in vivo tracking of biodistribution in reticuloendothelial organs of healthy Swiss Webster mice [238]. In another work, upconversion NPs, capable of converting NIR radiation to visible emission, were coated with RBC membrane radiolabeled with [^18^F] via an in vivo click chemistry method. This strategy enabled multimodal live imaging, combining MRI, upconversion luminescence imaging, and PET, in 4T1 triple-negative breast cancer-bearing mice [29]. Similarly, RBCs encapsulating the NIR fluorochrome ICG and carrying upconversion NPs on their surface were successfully employed for imaging-guided tumor surgery and PDT in HepG2 lymph node metastasis models [239]. Magnetic iron oxide NPs loaded with cypate, a NIR light-triggerable cyanine dye, and coated with RBC membranes enabled clear tumor visualization by T2-weighted MRI and enhanced tumor suppression through photothermal therapy in HCT-116 colorectal cancer-bearing mice [240]. More recently, RBC membranes functionalized with targeting ligands and encapsulating polymeric NP cores carrying both chemotherapeutic and imaging agents enabled specific tumor targeting, efficient doxorubicin delivery, and fluorescence imaging in MCF-7 breast cancer cells. This strategy demonstrated strong translational potential for personalized cancer therapy and offers flexibility to incorporate diverse imaging probes for multiple modalities [241]. RBC-based microrobots were developed by conjugating cypate to the RBC surface and encapsulating magnetic nanoparticles, the chemotherapeutic agent doxorubicin, and the antifibrotic drug pirfenidone within their cavities. Guided by an external magnetic field, this theranostic system accumulated at the tumor site and released its payload upon laser irradiation, for a highly efficient cancer therapy, which was monitored and confirmed in real time via in vivo fluorescence imaging in 4T1 tumor–bearing mice [242].

Similar to RBCs, macrophages are another promising theranostic carrier, owing to their innate tumor-homing capabilities. Macrophages can internalize mesoporous organosilica NPs encapsulating doxorubicin and perfluoropentane to create US-activatable carriers. Partial perfluoropentane vaporization produced an echogenic signal that enabled US-based monitoring of the enriched macrophages, while high-intensity focused US induced cavitation, triggering site-specific drug release [243] in 4T1 tumor-bearing mice. As well, a novel photothermal agent consisting of NP complexes containing polyvinylpyrrolidone, Fe^3+^ ions, and dopamine was loaded into macrophages, demonstrating PAI capability for image-guided photothermal treatment and highlighting strong potential for cancer treatment [244]. Macrophages were enriched with glucose oxidase nanozymes designed to sense the tumor microenvironment and induce starvation therapy, along with the fluorescent probe IR-820, which enables both NIR imaging and controlled drug release. In 4T1 tumor-bearing mice, this multifunctional system allowed real-time macrophage tracking, while IR-820-mediated local heating triggered the controlled release of glucose oxidase. This dual action effectively killed adjacent tumor cells and demonstrated strong potential for cancer therapy [245].

Neutrophils have also attracted interest as vessels for theranostics. Doxorubicin-loaded magnetic mesoporous silica NPs encapsulated within neutrophils enabled precise in vivo MRI tracking in U87 glioma-bearing mice. Following surgical resection, the targeted accumulation of chemotherapy agents at the tumor site effectively delayed tumor recurrence, as evidenced by longitudinal quantitative monitoring of T_2_-weighted signal intensity. Furthermore, ICG-labeled NPs provided complementary in vivo fluorescence imaging with comparable performance to MRI-based tracking [14]. In another approach, neutrophils were engineered with an RGD-apoptotic peptide conjugate (RA) and the photosensitizer Ce6. The selective delivery of the RA/Ce6 complex enabled a synergistic PDT and RA-induced mitochondrial disruption in melanoma and oral cancer models, with fluorescence monitoring confirming enhanced efficacy [246]. The strategy of activating apoptotic pathways was also achieved using neutrophil-derived exosomes loaded with caspase-triggering proteins and enriched with SPIONs for magnetically enhanced tumor targeting. In the same study, neutrophil-derived nanovesicles carrying doxorubicin, similarly decorated with SPIONs, enabled guided delivery under an external magnetic field. Both systems demonstrated efficient and safe drug delivery for cancer therapy, while NIR cyanine dye labeling enabled noninvasive in vivo monitoring of tumor accumulation [247]. In another work, hollow MnO_2_ NPs loaded with the cytotoxic porcine pancreatic elastase and the NIR dye IR780 were coated with neutrophil membranes and tested in a highly aggressive murine E0771 breast carcinoma model. Dual-modality MR/NIR imaging enabled real-time tracking of NP tumor accumulation and therapeutic response. The treatment enhanced antitumor efficacy after laser irradiation and improved mouse survival through synergistic apoptosis induction, ROS-mediated cytotoxicity, and T-cell activation [248]. Very recently, a neutrophil-based delivery platform, in which liposomes loaded with paclitaxel and transforming growth factor-β (TGF-β) siRNA were encapsulated within neutrophils, was applied in a murine non-small cell lung cancer model for synergistic chemo-immunotherapy. Labeling with the red fluorescent dye PKH26 confirmed tumor-specific biodistribution and enabled assessment of therapeutic response, highlighting its potential as a theranostic strategy [249].

In recent years, MSC-derived exosomes have gained prominence for tumor tracking, imaging, and therapy. MSC-derived exosomes functionalized with the Sgc8-c aptamer targeting protein tyrosine kinase 7 were employed as doxorubicin carriers and loaded with oxygen to enable ultrasonic imaging. In B16F0 melanoma-bearing mice, this theranostic system was successfully monitored via US, where contrast intensity reflected local accumulation and correlated with effective tumor growth suppression [37].

T cell–based systems, including CAR-T and T cell receptor (TCR)-engineered T cells, are important emerging tools for cancer therapy, highlighting the need for imaging techniques to noninvasively monitor their tumor accumulation and persistence in order to optimize efficacy and safety. To this end, CAR-T cells were labeled with ferumoxytol, an FDA-approved iron oxide nanoparticle formulation detectable by MRI, and evaluated in vivo in a murine osteosarcoma model. Ferumoxytol-labeled CAR-T cells were successfully visualized by multimodal MRI, PAI, and magnetic particle imaging (MPI), confirming osteosarcoma localization and highlighting the strong potential of this approach for clinical translation [250]. Similarly, TCR-transgenic and CAR-T cells were efficiently labeled with ultrasmall SPIONs and tested in a murine glioma model, allowing high-sensitivity longitudinal monitoring by MRI following intratumoral injection [251]. More recently, cytotoxic T cells targeting ovalbumin (OVA) were engineered to deliver doxorubicin-loaded gold nanorods and administered intravenously to mice bearing OVA-expressing melanomas. This system enabled a combination of immunotherapy, chemotherapy, and in vivo cell tracking via US/PA imaging, allowing detection of nanorod accumulation within tumor masses, as well as monitoring of tumor regression following the combined treatment [252].

Cell-based theranostics has also explored the use of platelets as a delivery platform. NPs co-encapsulating doxorubicin and the photothermal agent ICG were incorporated into platelet membranes and evaluated in both xenograft and orthotopic MDA-MB-231 mouse models of breast cancer. These multifunctional systems enabled targeted chemo-photothermal therapy that completely ablated primary tumors, inhibited metastasis and allowed noninvasive monitoring of tumor growth via fluorescence imaging [253]. In a more recent study, a platelet-mediated delivery system co-encapsulating the tyrosine kinase inhibitor dasatinib and the chemosensitizer atovaquone demonstrated enhanced therapeutic efficacy against liver cancer. Labeling these carriers with Cy5.5 enabled assessment of biodistribution in rodent models, showing efficient targeting of liver cancer tissues and increased drug accumulation at the tumor site [254].

Effective bioimaging and cell tracking are essential for evaluating the success of targeted delivery strategies. Collectively, these studies underscore the tangible potential of cell-based platforms to serve both as drug delivery vehicles and as imaging tools, enabling confirmation of selective tumor targeting and providing quantitative measures to assess therapeutic efficacy.

## 5. Conclusions

Cell-based drug delivery systems have long been viewed as a promising approach for targeted therapy in cancer. As highlighted across the studies reviewed, developments in chemistry, materials science, and bioengineering have gradually shifted the field from conventional cell carriers toward more advanced biomimetic micro- and nanoscale systems. By combining biological features with controlled motion and regulated drug-release mechanisms, these diverse platforms offer a level of spatial and temporal precision that was previously difficult to achieve, together with integrated diagnostic and therapeutic functionalities.

The literature examined in this review indicates that such innovations may hold particular relevance for central nervous system tumors, especially glioblastoma, where treatment options remain limited and outcomes are poor. Several recent attempts to deliver therapeutic agents across the blood–brain barrier have generated cautious optimism, particularly in approaches incorporating CAR-T-based strategies, which are now being explored in multiple clinical trials. However, the evidence also shows that achieving effective systemic delivery remains challenging. Many advanced interventions continue to rely on intracranial administration or local delivery supported by external reservoirs, and concerns regarding neurotoxicity have become increasingly prominent, with implications for patient safety and quality of life.

Overall, the collective findings suggest that further progress will depend on refining these delivery systems to improve safety, stability, and performance within the highly heterogeneous tumor microenvironment. Continued efforts in this direction will be essential to translate the promising concepts identified in current research into reliable and clinically meaningful treatments for glioblastoma and other difficult-to-treat cancers.

## Figures and Tables

**Table 2:** Recent development of brain-targeted cell-based drug delivery systems.

Type	Modification	Delivery	Agent/Drug	Disease	Refs.	Year
MSCs	Human MSCs	FUS	MCSs	Parkinson’s Disease	[96]	2025
	Human umbilical cord-derived mesenchymal stem cells (hUC-MSCs)	IV	Cytopeutics^®^ hUC-MSCs (Neuroncell-EX)	Ischemic Stroke	[97]	2025
	Muse (Multilineage Differentiating Stress Enduring) Cells	Nasal	Muse cells	Ischemic stroke	[98,99]	2025
	Umbilical cord mesenchymal stem cells (UMSCs) transport miR-124 and programmed cell death protein-1 (PD-1)	IC	UMSC/miR-124-PD-1 plasmid	Glioblastoma	[99]	2025
	Umbilical cord mesenchymal stem cells (UMSCs) using gadodiamide-concealed magnetic nanoparticles (Gd-FPFNP)	Magnetically	Gadolinium	Glioblastoma	[100]	2025
	Adipose-derived stem cells (ADSCs) with lentiviral transfection	IC	Lentiviral expression of alpha statina (Al-ADSC)	Glioma	[101]	2025
	Adipose-derived stem cells (ADSCs) with miRNAs and ultra-small paramagnetic nanoparticles (USPNs)	Nasal	hADSCs were transduced with Multi-miR-MUT	Traumatic brain injury	[102]	2025
	Human umbilical cord-derived mesenchymal stem cells (hUCMSCs)	Intrathecal	hUCMSCs	Spinal cord injury	[103]	2023
	Human umbilical cord-derived mesenchymal stem cells (hUCMSCs)	In vitro	Secretory factors HGF, BDNF, and TNFR1	Stoke-brain injury	[104]	2023
	Adipose-derived mesenchymal cells	IP	Th1/Th17 and expansion of Th2/Treg responses	Multiple sclerosis	[105]	2023
	Allogeneic twin stem cell (TSC) system composed of two tumor-targeting stem cell (SC) populations.	Locoregional	Acquire resistance to oHSV and release immunomodulators (GM-CSF)	Brain metastasis	[106]	2023
	Engineered MSCKO- IFNb to co-express scFv-PD1 (MSCKO-IFNb-scFv-PD1)	IC	(MSCKO-IFNb-scFv-PD1)	Glioblastoma	[107]	2023
Exosomes	CpG oligodeoxynucleotide-functionalized exosomes (Exo-CpG)	ID	GMB	Glioblastoma	[108]	2025
	Endothelial progenitor cell-derived exosomes (EPC-derived exosomes)	IV/LIPFUS	EPC	Stroke	[109]	2025
	Decellularized extracellular matrix gel-encapsulated exosomes (dECM@Exo)	IC	hUCMSC	Neuroinflammation	[110]	2025
	Macrophage-derived exosomes loaded with curcumin and methylene blue	IP	EXO-Cur + MB	Alzheimer’s disease	[111]	2025
	Mesenchymal stem cell-derived exosomes loaded with rapamycin (Exo-RAPA)	IV	EXO-RAPA	Glioblastoma	[112]	2025
	Glioblastoma cell-derived exosomes carrying bevacizumab (Exo-BEV)	IV	EXO-BEV	Glioblastoma	[113]	2025
	Exosomes loaded with a nondegradable form of IκB (Exo-srIκB)	In vitro	Exo-srIκB	Neuroinflammation/Aging	[114]	2025
	Exosomes engineered with RVG-Lamp2b-Irisin fusion protein	IP	Exos-RVG-Lamp2b-Irisin	Exertional heat stroke (EHS)	[115]	2025
	Mesenchymal stem cell-derived exosomes (MSC-derived exosomes)	Nasal	MSC	Subarachnoid hemorrhage	[116]	2025
	Bone marrow mesenchymal stem cell-derived exosomes (BMSCs-Exos)	Nasal	BMSC	Autoimmune encephalomyelitis	[117]	2025
	Exosomes co-loaded with BACE1 siRNA and Berberine	Nasal	MsEVB@R/siRNA	Alzheimer’s disease	[118]	2025
	Mesenchymal stem cell-derived small extracellular vesicles (MSC-sEVs)	In vitro	sEVs	Alzheimer’s disease	[119]	2025
	Human-induced pluripotent stem cell–derived neural stem cell exosomes (hiPSC-NSC-Exos)	Nasal	hiPSC-NSC-Exos	Intracerebral hemorrhage	[120]	2025
	M1-polarized macrophage-derived exosomes (M1 exosomes)	In vitro	MI	Glioblastoma	[121]	2025
	Sinomenine-treated microglia-derived exosomes	In vitro	SINO-EXO-miRNA-223-3p	Chronic cerebral hypoperfusion (CCH)	[122]	2025
	Virally infected endothelial progenitor cell-derived exosomes carrying HSP90 shSiRNA (EPC-Exos)	Nasal	m-eO-EPC-EXOs	Intracerebral hemorrhage	[123]	2025
	Human amniotic mesenchymal stem cell-derived anti-CD19 exosomes (anti-CD19-Exo)	IC	Anti-CD19-Exo-MTX	Central nervous system lymphoma (CNSL)	[124]	2025
	Sca-1^+^-selected multipotent progenitor cell–derived exosomes combined with intraspinal injection of neural stem cells (NSCs)	MSCs IV/NSCs IS	MPC	Spinal cord injury	[125]	2025
	Bone marrow mesenchymal stem cell–derived exosomes loaded with celastrol (BMSC-Exos-Cel)	IV	BMSC-EVs-Cel	Glioblastoma	[126]	2024
	Human umbilical mesenchymal stem cell-derived exosomes loaded with superparamagnetic iron oxide nanoparticles (HuMSC-Spion-Ex)	IV with magnetic targeting	SPION-Ex/MF	Post-stroke cognitive impairment (PSCI)	[127]	2024
	Folic acid-conjugated exosomes co-loaded with temozolomide (TMZ) and quercetin (Qct)	In vitro	TMZ-Qct-Exo-FA	Glioblastoma	[128]	2024
	Nanofibrous scaffold loaded with mesenchymal stem cells and neural stem cell–derived exosomes (Duo-Exo@NF)	IC	Duo-Exo@NF	Traumatic brain injury (TBI)	[129]	2024
Neural Stem Cells (NSCs)	dopaminergic neuron progenitors derived (hiPSCs) in a gel matrix with tacrolimus-loaded microparticles	IC	(hiPSCs)	Parkinson’s	[129]	2025
	Encapsulation of tumoricidal neural stem cells (NSCs) within an injectable chitosan (CS) hydrogel	IT	iNSCs) secreting (sTRAIL; sTR)	Glioblastoma	[130]	2024
	NSCs in biocompatible 3D hydrogel	In vitro	Neural stem cell (NSC)-containing scaffold	Neuronal diseases	[131]	2024
	Peripheral nerve-derived stem cell (PNSC) exhibiting Schwann cell-like phenotypes	Intrathecal	Peripheral nerve-derived stem cells (PNSCs)	Traumatic brain injury	[132]	2024
	iNSC-secreted RANTES/IL-15 enhancing chondroitin sulfate proteoglycan 4-targeted CAR-T cell	iNSCs-IC/CAR-T-IV	(CSPG4-CAR-T) activity/RANTES/IL-15	Glioblastoma	[133]	2023
RBCs	Red blood cell membrane-coated docetaxel drug nanocrystals modified with pHA-VAP (pV)	IV	Docetaxel (pV-RBCm-NC(DTX),	Glioma	[134]	2024
	Erythrocyte membrane (EM) functionalized with the tumor-penetrating peptide iRGD (CRGDK/RGPD/EC)	IV	(CRGDK/RGPD/EC)/Temozolomide (TMZ)	Glioblastoma	[135]	2024
	EM–coated polycaprolactone (PCL) nanoparticles (NPs) loaded with curcumin (Cur) and conjugated with TGNYKALHPHN (TGN)	IV	Curcumin (Cur)—TGN-RBC-NPs-Cur formulation	Alzheimer’s disease (AD)	[135]	2024
	Nano-erythrosomes	In vitro	Metformin (MET)	Glioblastoma	[136]	2023
	Erythro-Magneto-HA-Virosome (EMHV)	Magnetic	EMHV	Glioma	[137]	2020
Macrophages	Injectable oxidized high-amylose starch hydrogel (OHASM) containing macrophages	IT	Macrophages and BLZ945 (macrophage-polarizing drug)	Glioblastoma	[138]	2025
	Macrophages loaded with ferritin-conjugated monomethyl auristatin E (MDC)	In vivo	Ferritin-conjugated monomethyl auristatin E (MDC)	Glioblastoma	[139]	2025
	Engineered M2-like macrophages (eM2-Mφs)	In vitro	Engineered M2-like macrophages (eM2-Mφs)	Glioblastoma	[140]	2025
	Mitomycin-treated macrophages (Ma)/photosensitizer (PS)	IV	Photosensitizer-loaded macrophages (MaPS)	Glioblastoma	[141]	2024
GASC	PLGA nanoparticles (NPs) coated with GASC–glioma cell fusion (SG cell) membranes	IV	Temozolomide (TMZ)-loaded SGNPs	Glioblastoma	[142]	2023
	GASC-secreted CXCL14 promotes glioma cell invasion	In vitro	GASC-secreted CXCL4	Low-grade glioma	[143]	2018
Neutrophils	Mouse neutrophils (NE) loaded with hexagonal boron nitride nanoparticles carrying chlorin e6 (BNPD-Ce6)	IV/irradiation	BNPD-Ce6@NE	Glioblastoma	[144]	2025
	TMZ-loaded T7-cholesterol nanoparticle/neutrophils	IV	T7/TMZ-conveyed neutrophils (PMN/T7/TMZ)	Glioblastoma	[145]	2024
	NE- activated by Cyto-Adhesive Micro-Patches (CAMPs)	IP	NE/CAMPs combined with anti-programmed cell death-1 (aPD-1), termed *Checkmate 143*	Glioblastoma	[146]	2024
	Live neutrophils enveloping liposomes containing dexamethasone, ceftriaxone, and oxygen-saturated perfluorocarbon (Lipo@D/C/P)	IV	Lipo@D/C/P	Brain inflammation	[147]	2024
Outer Bacterial Membrane	OMVs carried small-interfering RNA (siRNA) and doxorubicin	In vivo	ΔmsbB OMVs + DOX + siCd47	Glioblastoma	[148]	2025
	Brain-tumor-seeking and serpin-inhibiting outer membrane vesicles (DE@OMVs)	IV	DE@OMVs (Dexamethasone/Embelin)	Metastatic glioblastoma	[149]	2024
	Pioglitazone encapsulation (PGZ)	In vivo	OMV@PGZ	Stroke	[150]	2023
	Doxorubicin (DOX)-loaded bacterial outer membrane vesicles (OMVs/DOX)	IV	Doxorubicin-loaded outer membrane vesicles (OMVs/DOX)	Glioma therapy	[151]	2023
	Lipopolysaccharide-free EC-K1 outer membrane	IV	dOMV@NPs	Glioblastoma	[152]	2022

FUS: Focus Ultrasound mediated; IV: intravenous; IC: intracranial; IP: intraperitoneal; ID: intradermal; IT: intratumoral.

**Table 3:** Recent CAR-T-based clinical trials.

Clin. Trial Id.	Modification	Delivery	Agent/Drug	Disease
NCT05168423	Bivalent (CAR) T cells targeting epidermal growth factor receptor (EGFR) epitope 806 and interleukin-13 receptor alpha 2 (IL-13Rα2)	Intrathecal	CART-EGFR-IL13Rα2	EGFR-amplified recurrent glioblastoma
NCT02208362	IL-13 cytokine-directed CAR mutated at a single site (E12Y) and incorporating a 4-1BB costimulatory domain	ICV	CART-EGFR-IL13Rα2	Glioblastoma
NCT05660369	(CAR) T cells targeting epidermal growth factor receptor (EGFR) variant III tumor-specific antigen, as well as the wildtype EGFR protein	ICV	CARv3-TEAM	Glioblastoma
NCT06815029	IL13Rα2-targeting chimeric antigen receptor (CAR) T cells with CRISPR knockout of TGFβR2	IC	TGFβR2KO/IL13Rα2-CAR T	IDH-mutant astrocytoma, grade 3/4
NCT06482905	Anti-B7-H3 CAR-T cell injection (Tx103).	IV	TX103	Recurrent progressive grade 4 glioma
NCT06186401	Anti-EphA2/IL-13Rα2 CAR (E-SYNC) T cells	IV	E-SYNC T	EGFRvIII-positive glioblastoma
NCT04185038	Autologous CD4+ and CD8+ T cells lentivirally transduced to express a B7-H3-specific chimeric antigen receptor (CAR)	ICV	B7-H3 CAR-T	Diffuse intrinsic pontine glioma (DIPG)
NCT05835687	Autologous B7-H3-CAR T cells.	OR	Loc3CAR	Primary CNS tumors
NCT05577091	The autologous Tris-CAR-T cell, targeting both CD44 and CD133, the two inversely correlated targets, with truncated IL7Ra	OR	Tris-CAR-T	Glioblastoma
NCT05366179	(CAR) T cells expressing B7-H3-specific chimeric antigen receptors	ICV	CAR-B7-H3T	Glioblastoma
NCT05353530	IL-8 receptor-modified CD70 CAR T cells	IV	8R-70CAR	CD70-positive adult glioblastoma
NCT03726515	EGFRvIII-directed CAR T cells and PD-1 inhibition	Infusion	CART-EGFRvIII T and Pembrolizumab	MGMT-unmethylated glioblastoma
NCT03283631	EGFRvIII CAR-T cells	CED	EGFRvIII-CAR	Glioblastoma
NCT02664363	EGFRvIII CARs	IV	EGFRvIII CARs	Newly diagnosed grade IV malignant glioma
NCT01109095	CD28 attached to the HER2 chimeric receptor (HER2-CAR)	IV	HER2-CD28 CMV-T cells	Glioblastoma multiforme
NCT07209241	CART-EGFR-IL13Rα2	Dosing schedules	CART-EGFR-IL13Rα2	EGFR-amplified glioblastoma
NCT07193628	EPC-003 Fully Human Anti-B7H3/IL13Ra2 Armored CAR-T Cell Therapy	OR	EPC-003 CAR-T	Refractory glioblastoma and recurrent glioblastoma
NCT07180927	Delta-like ligand 3 (DLL3)-specific CAR-T cells	IV	DLL3-CAR-T	DLL3-positive brain tumors, including glioblastomas and diffused intrinsic pontine or midline gliomas
NCT06815432	Chimeric antigen receptor (CAR) derived from an antibody called GC33	Dosing schedules	GC33-CAR-T	GPC3-positive brain tumors
NCT06691308	WL276 CAR-T cells	IC	WL276 CAR-T	Recurrent glioblastoma
NCT07209241	CART-EGFR-IL13Rα2	IC	SNC-109 CAR-T	EGFR-amplified recurrent glioblastoma
NCT05835687	B7-H3-CAR with a CD28z signaling domain and 41BB ligand (B7-H3-CAR T cells	Locoregional	B7-H3 CAR-T	Diffuse midline glioma
NCT05802693	EGFRvIII CAR-T	ICV	EGFRvIII CAR-T	Recurrent glioblastoma
NCT05474378	B7-H3 chimeric antigen receptor T cells (B7-H3CART)	ICV/IT	B7-H3-CART	Recurrent glioblastoma
NCT05063682	EGFRvIII CAR-T cells (EGFRvIII-specific hinge-optimized CD3ζ-stimulatory/41BB-co-stimulatory chimeric antigen receptor autologous T-lymphocytes)	ICV	EGFRvIII-CAR T	Leptomeningeal disease from glioblastoma
NCT04385173	Safety and efficacy of B7-H3 CAR-T therapy between temozolomide cycles	OR	B7-H3-CART	Refractory glioblastoma
NCT04214392	Chlorotoxin (EQ)-CD28-CD3ζ-CD19t-expressing CAR T lymphocytes (NCI SYs)	IT/ICV	(EQ)-CD28-CD3zeta-CD19t-CAR T	Recurrent MPP2-positive glioblastoma
NCT04045847	CD147-CAR-T cells	OR	CD147-CART	Recurrent glioblastoma
NCT04003649	IL13Rα2-CAR T cells (IL13Rα2-specific hinge-optimized 4-1BB costimulatory CAR/truncated CD19-expressing autologous TN/MEM cells)	ICV/IT	IL13Rα2-CAR T	Resectable recurrent glioblastoma

ICV: intraventricular; IC: intracranial; IV: intravenous; OR: Ommaya reservoir; CED: convection-enhanced delivery; IT: intratumoral.

## Data Availability

No new data were created or analyzed in this study.

## Abbreviations

The following abbreviations are used in this manuscript:

ACT	Adoptive cell transfer
BBB	Blood–brain barrier
CAR	Chimeric antigen receptor
CED	Convection-enhanced delivery
CNS	Central nervous system
CSFB	Cerebral spinal fluid–blood barrier
CT	Computed tomography
DDS	Drug delivery system
EPR	Enhanced permeability and retention
GASCs	Glioma-associated stem cells
GBM	Glioblastoma
ICG	Indocyanine green
MC	Macrophages
MPS	Mononuclear phagocytic system
MRI	Magnetic Resonance Imaging
MSC	Mesenchymal stem cell
NETs	Neutrophil extracellular traps
NIR	Near Infrared
NK	Natural Killer cells
NPs	Nanoparticles
PAI	Photoacoustic Imaging
PET	Positron Emission Tomography
PLGA	Polylactic-co-glycolic acid
RBC	Red blood cell
SPIONs	Superparamagnetic iron oxide nanoparticles
TAMs	Tumor-associated macrophages
TIL	Tumor-infiltrating lymphocytes
TMZ	Temozolomide
US	Ultrasound
